# Terpene Composition Complexity Controls Secondary Organic Aerosol Yields from Scots Pine Volatile Emissions

**DOI:** 10.1038/s41598-018-21045-1

**Published:** 2018-02-14

**Authors:** C. L. Faiola, A. Buchholz, E. Kari, P. Yli-Pirilä, J. K. Holopainen, M. Kivimäenpää, P. Miettinen, D. R. Worsnop, K. E. J. Lehtinen, A. B. Guenther, A. Virtanen

**Affiliations:** 1 Department of Applied Physics, University of Eastern Finland, P.O. Box 1626, 70211 Kuopio, Finland; 2 Department of Ecology and Evolutionary Biology, University of California Irvine, Irvine, CA 92697-2525 United States; 3 Department of Environmental and Biological Sciences, University of Eastern Finland, P.O. Box 1627, 70211 Kuopio, Finland; 4 Aerodyne Research Inc., Billerica, Massachusetts 08121-3976 United States; 5 Department of Physics, University of Helsinki, P.O. Box 64, 00014 Helsinki, Finland; 6 Finnish Meteorological Institute, Kuopio, PO Box 1627, 70211 Kuopio, Finland; 7 Department of Earth System Science, University of California, Irvine, CA 92697-3100 United States

## Abstract

Secondary organic aerosol (SOA) impact climate by scattering and absorbing radiation and contributing to cloud formation. SOA models are based on studies of simplified chemical systems that do not account for the chemical complexity in the atmosphere. This study investigated SOA formation from a mixture of real Scots pine (*Pinus sylvestris*) emissions including a variety of monoterpenes and sesquiterpenes. SOA generation was characterized from different combinations of volatile compounds as the plant emissions were altered with an herbivore stress treatment. During active herbivore feeding, monoterpene and sesquiterpene emissions increased, but SOA mass yields decreased after accounting for absorption effects. SOA mass yields were controlled by sesquiterpene emissions in healthy plants. In contrast, SOA mass yields from stressed plant emissions were controlled by the specific blend of monoterpene emissions. Conservative estimates using a box model approach showed a 1.5- to 2.3-fold aerosol enhancement when the terpene complexity was taken into account. This enhancement was relative to the commonly used model monoterpene, “α-pinene”. These results suggest that simplifying terpene complexity in SOA models could lead to underpredictions in aerosol mass loading.

## Introduction

Plants are the largest source of volatile organic compound (VOC) emissions globally^1^. After entering the atmosphere, plant volatiles undergo oxidative chemistry that generate low-volatility vapors and contribute to the production of biogenic secondary organic aerosol (SOA)^2,3^. Atmospheric aerosols affect climate by scattering and absorbing radiation and by contributing to cloud-formation processes^4,5^. Potential climate feedbacks resulting from this type of biosphere-atmosphere interaction have been targeted as a high priority area for the future of atmospheric chemistry research^6^. However, there are many challenges to improving biogenic SOA predictive capabilities. One of these challenges is addressing the chemical complexity of plant VOC emissions. More than 1,700 compounds have been identified from plant volatile emissions^7^ and detailed SOA chemistry has only been investigated in laboratory chambers for a small fraction of these. The objective of this research was to use real Scots pine emissions to identify chemical controls on SOA production from oxidation of a natural, complex VOC mixture.

The chemical complexity of plant volatile emissions must be simplified in regional and global models due to the limited number of observations and the computational expense of including additional compounds. There are several approaches for doing this. Some models predict SOA as a prescribed source based on previous estimates of SOA mass loadings^8^. Other models partition reacted vapors from the gas-phase to the particle-phase assuming a single SOA yield. For example, the Norwegian Earth System Model (NorESM) assumes a 15% SOA yield from all monoterpenes^9^. Others add more complexity using absorption-partitioning theory where SOA mass yields are higher with increasing condensed phase organic mass to enhance absorption^10^. Widely-used implementations of absorption-partitioning theory include the Odum two-product approach^11^ or, more recently, the volatility basis set approach^12,13^. However, models that account for absorption effects are based on parameters derived from simplified chemical systems in laboratory chambers that do not represent the chemical complexity of real plant emissions.

Natural chemical complexity of VOC emissions presents an obstacle for predicting both current and future SOA production. Climate plays a key role in maintaining ecosystem health—including plant health. For example, moderate wintertime temperatures decrease insect mortality leading to increased severity of herbivore outbreaks in the spring^14^. When faced with indirect climate stressors such as this, plant defense responses are activated which alter the composition and magnitude of VOC emissions^15,16^. Plant volatile responses to herbivore stress are highly variable and depend on several factors including the degree of damage^16^, type of plant species^17^, herbivore species^16,18^ and presence of multiple interacting stressors^15,19–21^. Many herbivores increase the release of constitutively-emitted terpenoid compounds, such as monoterpenes and sesquiterpenes, that were emitted at a lower level prior to the stress. Alternatively, the stress can induce *de novo* biosynthesis and subsequent emission of other terpenoid compounds, or entirely different compound types, such as plant stress hormones including methyl jasmonate or methyl salicylate^22^. Other plant stress emissions are derived from mechanical damage. For example, defoliators damage cell membranes which subsequently degrade and lead to emissions of six-carbon green leaf volatiles such as 1-hexenol and hexenyl acetate^23^. Consequently, the volatile bouquet of plant emissions under herbivore stress contains a complex mixture of organics—many of which can act as precursors to SOA formation and many of which have never been considered in chamber SOA studies.

A few studies have demonstrated that plant stress VOCs can contribute to SOA production^24–28^. However, these studies did not account for absorption-partitioning effects in their experimental design. This gap limits data interpretation for determining the chemical controls on SOA production from a mixture of plant volatile emissions and how those chemical controls change within different plant volatile mixtures. To address this critical gap, we used emissions from Scots pine saplings to generate SOA directly from a complex mixture of plant volatiles before, during and after a pine weevil herbivore stress treatment across a range of overlapping organic aerosol mass loadings using an oxidation flow reactor. Scots pine (*Pinus sylvestris L*.) (Pinaceae) is one of the most widely distributed conifer tree species in the world^29^ and comprises 65% of the whole forest area in Finland^30^. The distribution of Scots pine covers Eurasian conifer forests from Scotland to Eastern Siberia close to the Pacific Ocean and south to Turkey. Thus, it is the most representative conifer species of boreal forests. This experimental design allowed us to investigate SOA production from a variety of different complex VOC mixtures representing unstressed emission profiles and herbivore-stressed emission profiles. Importantly, the goal of this research was not necessarily to infer the effects of pine weevil stress on SOA production in the boreal forest environment directly, but rather the goal was to investigate the chemical controls on SOA production as the mixture changes. This is an important distinction because improved understanding of the specific chemical controls on SOA generation is transferrable to other studies even if the plant emission response is different. By directly characterizing the impact of changing emissions on SOA production we seek to provide a framework for understanding the SOA implications from the many plant stress emissions studies reported previously.

**Figure 1:**
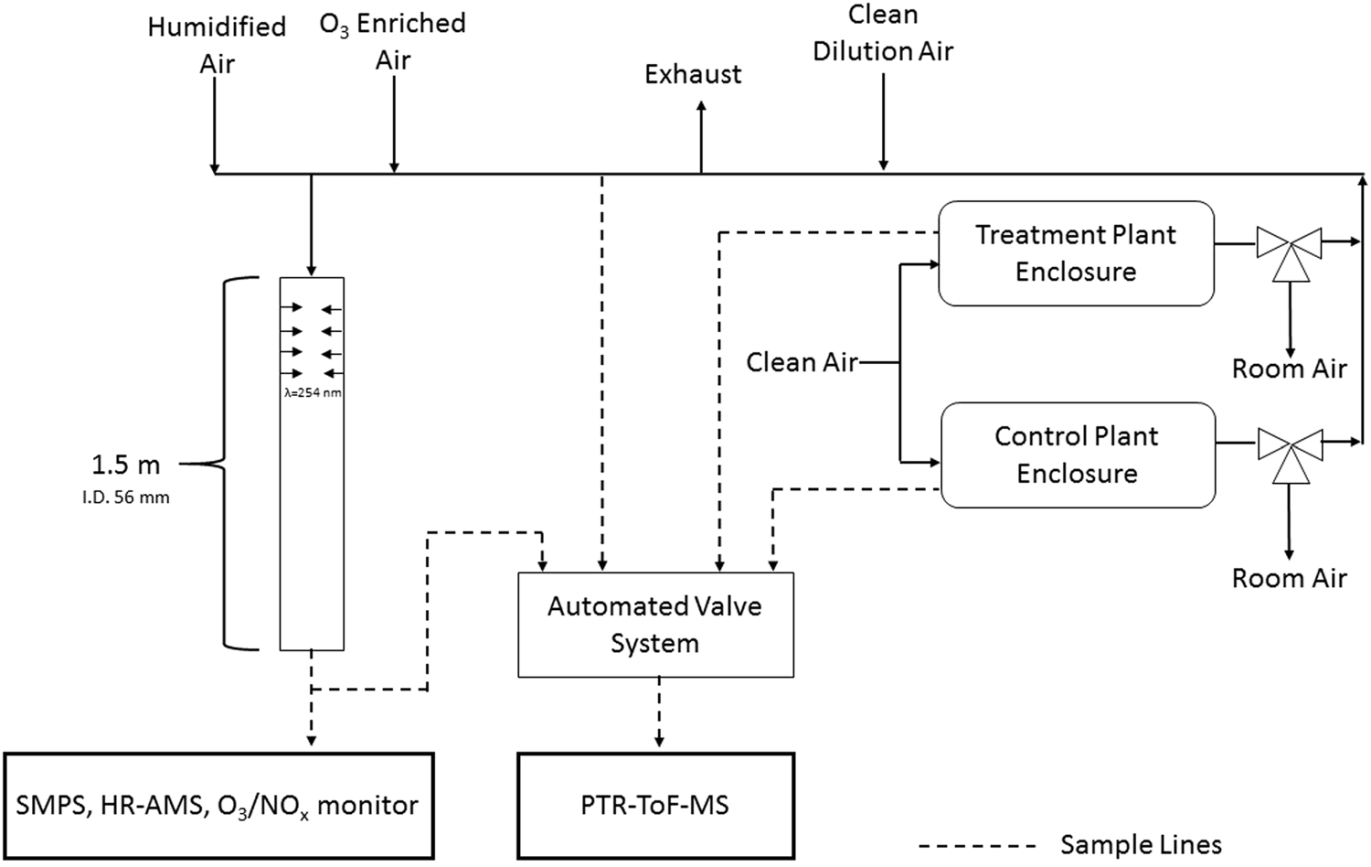
Flow reactor design for studying aerosol production from real plant volatile emissions. Humidified air and ozone-enriched air were added to air from the plant enclosure before entering the flow reactor. Three-way valves were located downstream of the plant enclosures to select which plant volatiles were used in aerosol experiments. Clean dilution air was added to adjust the VOC concentration to measure aerosol yields across a range of aerosol mass loadings.

A schematic of the SOA generation system used in this study is provided in Fig. 1. This study represents the most comprehensive investigation of the major chemical controls on SOA production from real plant emissions made possible by the high throughput of the flow reactor technique and the novel study design that accounted for important absorption effects. SOA from Scots pine emissions was compared to SOA generated from oxidation of individual monoterpene and sesquiterpene standard compounds. SOA mass yields from Scots pine emissions were controlled by the composition of the terpene emissions; both the sesquiterpene emission rate and the mixture of monoterpene isomers could explain the observed yields. These results suggest that over-simplifying the terpene complexity in SOA models could lead to significant underpredictions of SOA in forest environments.

## Results

We characterized SOA production from different mixtures of plant volatiles using a plant-herbivore system that included seven-year-old Scots pine (*Pinus sylvestris*) saplings before, during and after a 48-hour exposure to pine weevils (*Hylobius abietis*). SOA was generated via photooxidation of plant volatile emissions in an oxidation flow reactor (OFR). Table 1 summarizes the dates and duration for each of the four experiments conducted in summer 2015 with dates and time provided for herbivore application and removal. The number of averaging intervals used to characterize SOA generation from each experimental phase (pre-treatment, active feeding and post-treatment) is also provided. The averaging interval for SOA characterization was defined as the start of the plant volatile measurement at the OFR inlet to the end of the plant volatile measurement at the OFR outlet using proton transfer reaction time-of-flight mass spectrometry (PTR-ToF-MS). The “active feeding” experimental phase was defined based on an observable plant emission response using the PTR-ToF-MS data (see Supplementary Information Fig. 1 for example). Each experiment included one treatment plant and one control plant. The control plant was used to characterize emission variability to determine if any emission changes observed after treatment were due to the herbivore stress.

**Table 1:** Summary of SOA generation experiments conducted using Scots pine saplings during June-July of summer 2015.

Exp Number	Experiment Duration	Herbivore Application	Herbivore Removal	Number of SOA Averaging Intervals		
				Pre-Treatment	*Active Feeding	Post-Treatment
1	June 1–June 12	June 8 14:40	June 10 13:30	12	10	9
2	June 22–July 3	June 29 11:30	July 1 13:30	0	6	33
3	July 3–July 16	July 8 12:00	July 10 16:00	1	21	109
4	July 16–July 31	July 22 11:00	July 24 15:15	16	35	47

^*^Active feeding refers to periods where the PTR-ToF-MS measurements indicated there was a plant emission response. Data points collected after herbivores were applied but before a response was detected were included in the “pre-treatment” experiment phase.

### Plant Emission Rates Before, During and After Pine Weevil Feeding

**Figure 2:**
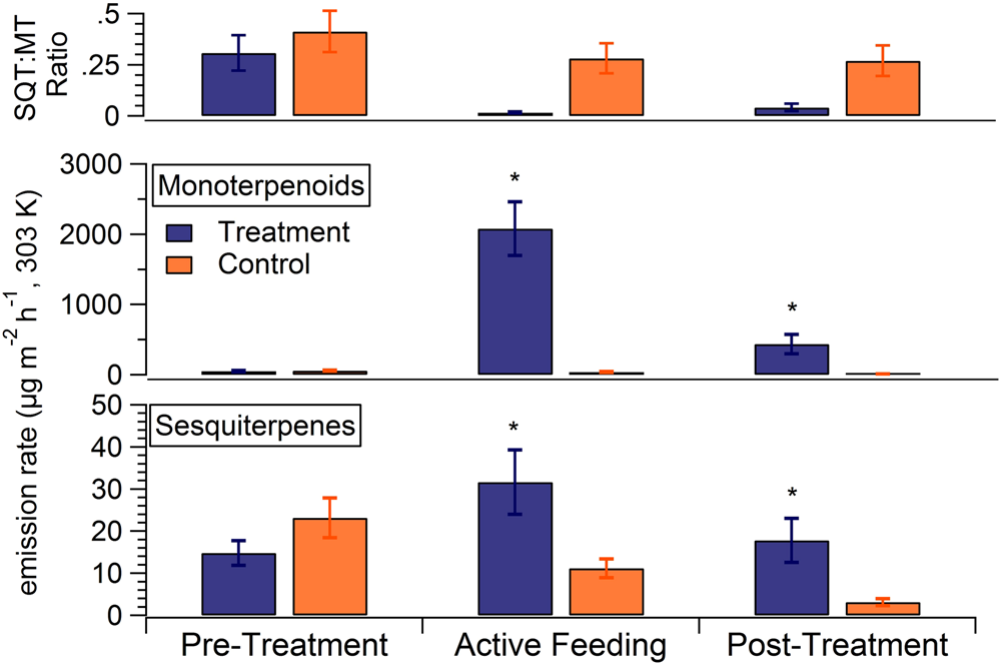
Summary of plant volatile emissions before, during and after pine weevil herbivory. The average monoterpenoid (middle panel) and sesquiterpene (bottom panel) emission rates during the three experimental phases. The top panel shows the sesquiterpene (SQT) to monoterpene (MT) emission ratio. Asterisks indicate significant differences between control and treatment plant emission rates (t-test, p < 0.05).

A summary of the average monoterpene and sesquiterpene emission rates in each experimental phase (pre-treatment, active feeding and post-treatment) is illustrated in Fig. 2. On average, monoterpene emissions from the treatment plant increased from 48.1 +/− 9.8 µg m^−2^ h^−1^ before treatment to 2080 +/− 383 µg m^−2^ h^−1^ during active feeding. For comparison, the control plant monoterpene emissions were 56.1 +/− 7.8 µg m^−2^ h^−1^ and 39.7 +/− 6.7 µg m^−2^ h^−1^ during pre-treatment and active feeding experimental phases, respectively. Sesquiterpene emissions from the treatment plant increased from 14.8 +/− 2.9 µg m^−2^ h^−1^ before treatment to 31.7 +/− 7.7 µg m^−2^ h^−1^ during active feeding.

**Figure 3:**
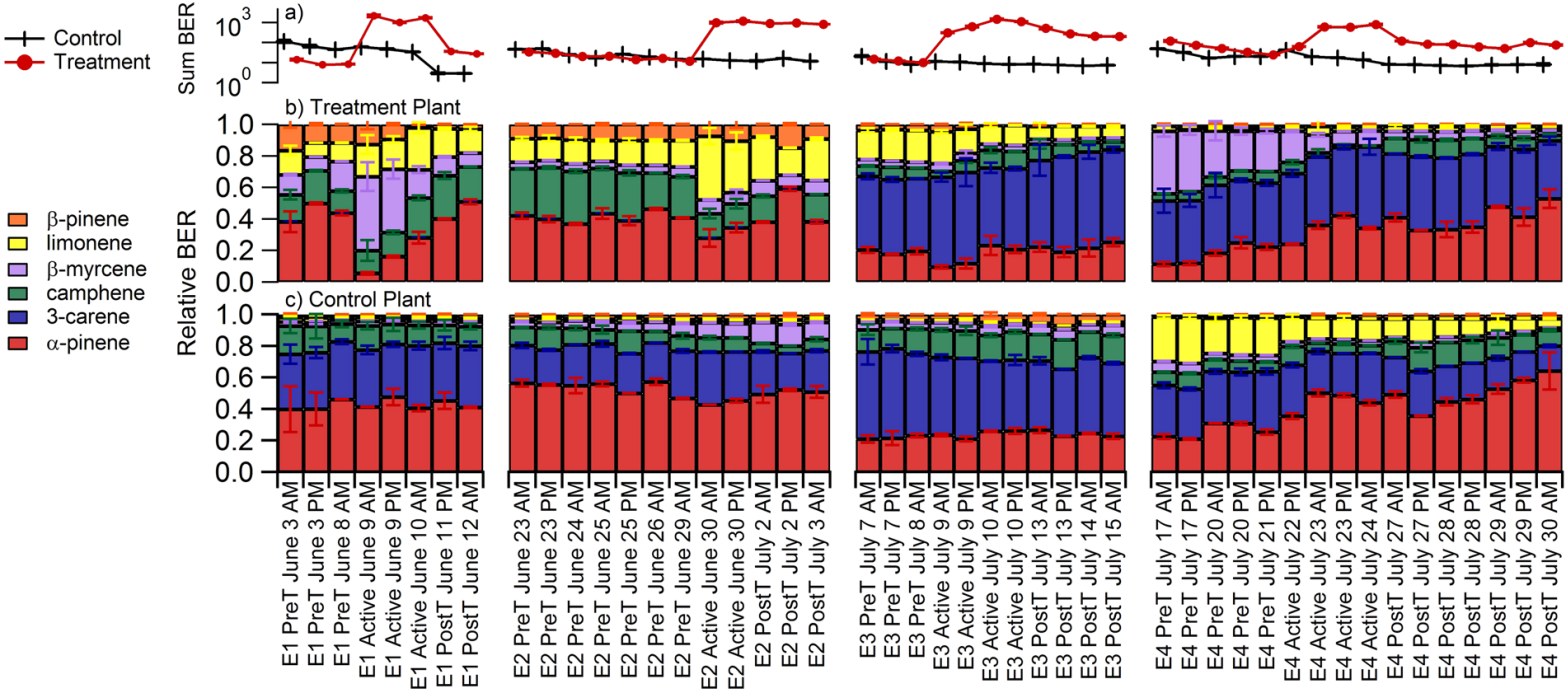
Detailed characterization of monoterpene emission profiles from all four experiments. The x-axis labels indicate the experiment number (E1 = experiment 1, E2 = experiment 2, …), experimental phase (PreT = pre-treatment, Active = active feeding, PostT = post-treatment), date and time of each duplicate cartridge sample collection. Panel (a) shows a time-series of the sum basal emission rate (BER) for the six dominant, quantitative monoterpene emissions identified in the cartridge samples on a log scale. Units are µg m^−2^ h^−1^. Panels (b) and (c) show the relative emission profile from the treatment plant and control plant, respectively. Error bars denote the standard deviation of duplicate samples from each seedling.

Sesquiterpene emissions from the control plant were 23.1 +/− 4.7 µg m^−2^ h^−1^ and 11.2 +/− 2.2 µg m^−2^ h^−1^ for the pre-treatment and active feeding experimental phases. Post-treatment, monoterpene and sesquiterpene emissions from the treatment plant were 435.5 +/− 139.1 µg m^−2^ h^−1^ and 17.8 +/− 5.2 µg m^−2^ h^−1^. Both emission rates for the post-treatment periods were lower than during the active feeding period, but remained elevated above pre-treatment emission rate values. Furthermore, they were higher than post-treatment control emissions with statistical significance for both monoterpene and sesquiterpene emission rates. Monoterpene and sesquiterpene emissions from treatment plants were significantly different from control emissions during active feeding and post-treatment (t-test). The sesquiterpene emissions from the control plants exhibited a gradual decrease over the length of each experiment. This is likely not due to a seasonal effect because each of the four experiments was conducted at different stages of growth from early summer to late summer. Rather, this gradual decline in emissions indicates that conditions in the laboratory enclosures (including water vapor concentrations and light intensity) were not ideal for maintaining plant photosynthesis over time. This is also evident in Fig. 3 and is discussed further in the next section. Pre-treatment monoterpene and sesquiterpene emissions were not statistically different between control and treatment plants.

A t-test was used to test for significance between observed differences in emission rates between the treatment and control plants during the different experimental phases (Fig. 2). The number of data points used to calculate the averages presented in Fig. 2 were as follows: pre-treatment (n = 20), active feeding (n = 15) and post-treatment (n = 18). Statistical significance is indicated in Fig. 2 for differences in monoterpenoid emission rate between treatment and control during active feeding (combined effective degree of freedom = 14.01, t-value = 5.329) and post-treatment (combined effective degree of freedom = 17.00, t-value = 3.048). There was no significant difference between control and treatment plants pre-treatment (combined effective degree of freedom = 36.2, t-value = −0.6402). Similarly, for sesquiterpene emissions there were significant differences between treatment and control plants during active feeding (combined effective degree of freedom = 16.32, t-value = −2.5753) and post-treatment (combined effective degree of freedom = 17.87, t-value = −2.7646). There was no significant difference between control and treatment plants pre-treatment (combined effective degree of freedom = 31.78, t-value = 1.5036).

Monoterpene and sesquiterpene emissions increased significantly for the treatment plants compared to the control group. Specifically, monoterpene emissions increased by 4000% while sesquiterpene emissions increased by 114%. The top line of Fig. 2 highlights the larger herbivore effect on monoterpene (MT) emission rates compared to sesquiterpene (SQT) emission rates in the treatment plants with SQT:MT emission ratios of 0.31, 0.02 and 0.04 for pre-treatment, active feeding and post-treatment periods respectively. The control group SQT:MT emission ratios were 0.41, 0.28 and 0.27 for the same respective periods. These unstressed SQT:MT ratios are within the range of reported field measurements from different Scots pine chemotypes where SQT:MT ratios ranged from 0–0.5^31^. We note this herbivore effect on plant emissions is in the opposite direction compared with that observed by Zhao and colleagues^28^ from aphid herbivory where they measured unstressed, intermediate stressed and maximum stressed SQT:MT from boreal forest trees of 0.119, 0.365 and 3.125, respectively. This is probably due to the different plant stress response mechanisms to chewing (e.g. beetles) and sucking (e.g. aphids) herbivore types. Constitutive (or unstressed) emissions are derived from a combination of *de novo* and pool emission sources^32^. Aphid herbivory induces *de novo* synthesis of new compounds that are subsequently emitted. In contrast, the bark-boring pine weevils used in this study mechanically damaged the plant bark which exposed terpene pools to the atmosphere. This mechanical damage likely led to a larger increase in emissions sourced from terpene pools than from *de novo* emissions. In a Scots pine forest environment, *de novo* monoterpene emissions contribute 30–46% of total monoterpene emissions with the remaining sourced from the pools^33^, but this has not been quantified for sesquiterpene emissions. Our results demonstrated a much larger increase in monoterpene emissions than sesquiterpene emissions after terpene pools were exposed to the atmosphere. This could suggest that a larger percentage of constitutive sesquiterpene emissions were derived from *de novo* synthesis. An alternate explanation could be that MT-rich resin was conducted from xylem and needles to the wounds for healing.

### Characterization of Plant Volatile Emission Profiles Used for SOA Generation

Figure 3a–c shows a sub-set of the GC data to demonstrate the variation in plant responses and emission profiles that were used for SOA generation. The compounds included in Fig. 3 are all monoterpenes that were characterized with an authentic GC standard and constituted greater than 5% of total emissions by mass during at least one of the experimental phases. These compounds were α-pinene, Δ-3-carene, camphene, β-myrcene, limonene and β-pinene. Two other compounds, p-cymene and β-phellandrene, comprised a major portion of the total emissions, but we did not have analytical standards for those compounds and thus their results are semi-quantitative. All quantitative and semi-quantitative emission rates measured from cartridge samples and averaged from each experimental phase for all four experiments are provided in the supplementary information (Supplemental Table 1). Figure 3a shows the sum of the basal emission rate (BER, normalized to 303 K, µg m^−2^ h^−1^) from these six compounds on a log scale. Each experiment demonstrates an evident increase in monoterpene emissions during active feeding. During experiments 1, 3 and 4 the emission rate decreased after herbivore removal, but stayed elevated above pre-treatment emissions rates. The treatment plant in experiment 2 maintained high emission rates after herbivores had been removed. The sum BER from the control plants decreased over time. This further highlights the strong herbivore effect on emission rates because the gradual reduction in control plant emission rates suggests that laboratory lighting was not sufficient to maintain photosynthesis rates.

Figure 3b and c illustrate the relative emission profiles from the treatment and control plants, respectively. There was clear variation between tree chemotypes used in this study^34^. This was evident by comparing the treatment and control plant emissions from experiments 1 and 2; the control plants were 3-carene emitters while the treatment plants were not and the treatment plants emitted an appreciable amount of limonene while the control plants did not. There was also variation in which compounds were most affected by herbivore treatment during the different experiments. For example, in experiment 1 the active feeding period corresponded to an increase in the relative β-myrcene emissions and, to a lesser extent, limonene emissions. Alternatively, active feeding during experiment 2 led to an increase in the relative limonene emissions, but not the β-myrcene emissions. In experiment 4, β-myrcene was emitted in a higher proportion before herbivore treatment than it was during or after herbivore treatment. It is likely that the plant emission profile before, during and after treatment was influenced by a host of factors including plant chemotype, seasonal variation throughout the summer and phenotype plasticity in responding to the herbivore stress. Even though there was no single “universal stress compound” observed in each of the four pseudo-replicate experiments, one consistent result was the SQT:MT ratio decreased by an order of magnitude from pre-treatment to active feeding (recall Fig. 2).

Figures 2 and 3 clearly demonstrate that emission profiles varied dramatically from experiment to experiment and between unstressed and stressed plants. This provided a unique opportunity to investigate factors controlling SOA production under varying monoterpene emission profiles and under different SQT:MT emission ratios.

### SOA mass yields from Scots pine volatiles

**Figure 4:**
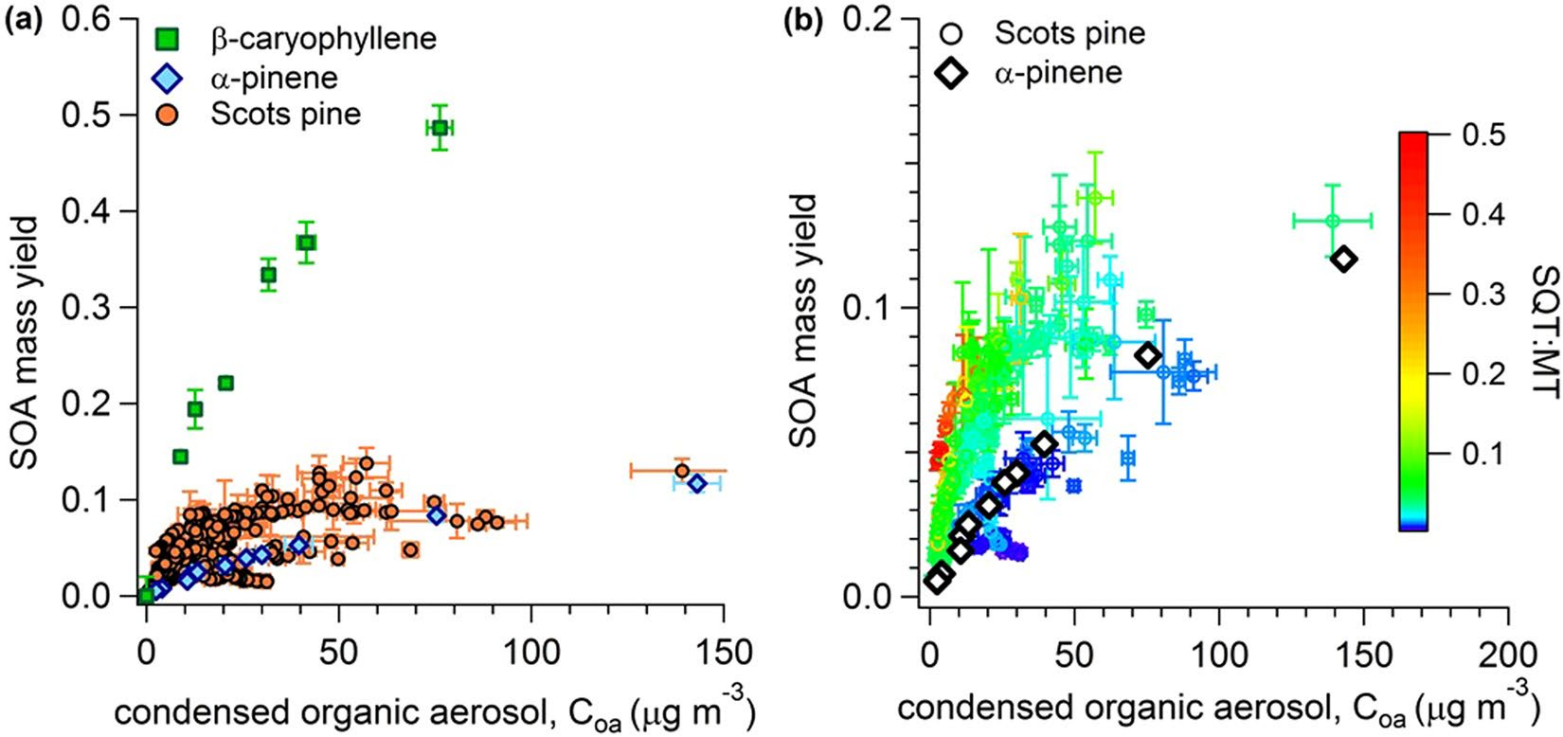
SOA mass yields from Scots pine volatile emissions. (**a**) Mass yields of SOA generated from Scots pine emissions during all experiments and all experimental phases compared to SOA generated from a monoterpene standard (α-pinene) and a sesquiterpene standard (β-caryophyllene). (**b**) Mass yields of SOA generated from Scots pine emissions from all four experiments and all experimental phases colored by the sesquiterpene (SQT) to monoterpene (MT) ratio at the flow reactor inlet. SOA mass yields from α-pinene are shown again for comparison.

A summary of all SOA mass yields measured from Scots pine VOC emissions for all experiments is shown in Fig. 4a. For comparison, SOA mass yield curves from two standard compounds are also shown—a monoterpene, α-pinene and a sesquiterpene, β-caryophyllene. The α-pinene SOA mass yields range from 0.5% to 12% across a condensed organic aerosol mass range of 2.5–143 µg m^−3^ in this OFR. These yields are lower than yields from α-pinene that have been presented in the literature previously, where yields have been reported as high as 40%^35–40^. These reduced yields can be attributed to vapor deposition of VOC oxidation products on the OFR walls. We characterized vapor deposition in the OFR used in this study by measuring SOA mass yields from standard compounds at different seed concentrations and found that yields were strongly dependent on particle seed surface area in this OFR (Supplementary Figure 2). This demonstrates substantial loss of low volatility vapors to the flow reactor walls. This phenomonon could also be attributed to uptake/partitioning kinetic limitations within the OFR due to the very short residence time. Regardless of mechanism, this means all yields presented here are underestimated compared to yields measured without these limitations, but does not prevent us from studying relationships between SOA precursors and SOA mass yields which can be used to investigate implications for relative changes in SOA production in forest environments. Furthermore, these results are the most comprehensive investigation directly linking complex plant emissions to SOA production efficiency while accounting for absorption effects.

In general, SOA mass yields from Scots pine emissions were higher than SOA mass yields from α-pinene alone (Fig. 4a). Most SOA yields from Scots pine emissions were between the yields from α-pinene and β-caryophyllene standards suggesting both monoterpenes and sesquiterpenes contributed to SOA mass. Figure 4b shows the same Scots pine data points, but now colored by the relative SQT:MT emission ratio (measured with PTR-ToF-MS). PTR-ToF-MS sesquiterpene measurements were corrected for sampling line wall losses based on an inter-comparison between the GC and PTR-ToF-MS measurements (see Supplementary Figure S3 for more details). Higher yields were correlated with higher SQT:MT ratios. The highest SQT:MT ratios were observed from unstressed plants before herbivore treatment representing typical emissions from healthy Scots pines in boreal forests. When the SQT:MT ratio was less than 0.025 (dark blue and purple points), the SOA mass yield curve was similar to the curve generated from oxidation of the α-pinene standard. This low SQT:MT ratio was observed during active feeding during Experiments 3 and 4. When SQT:MT ratios were greater than 0.05, the SOA yields were higher than α-pinene alone and the yield increased with increasing SQT:MT. These results demonstrate that sesquiterpenes played an important role in defining the overall SOA mass yield from Scots pine emissions.

### Theoretical Estimates of SOA Mass Yields

**Figure 5:**
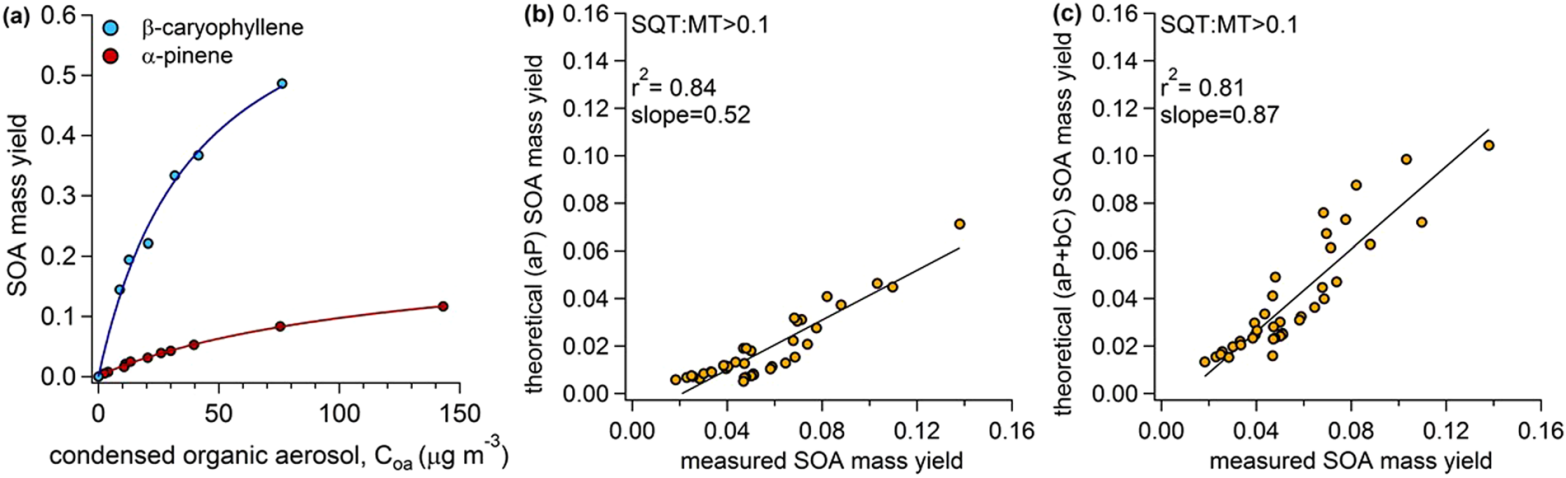
Odum Theoretical and observed SOA mass yields from healthy, unstressed Scots pine volatile emissions. (**a**) Odum fits for the two standard terpene compounds, α-pinene fits were α_1_ = 0.003, α_2_ = 0.218, k_1_ = 0.226, k_2_ = 0.008; β-caryophyllene fits were α_1_ = 0.735, α_2_ = 0, k_1_ = 0.025, k_2_ = 0. (**b** and **c**) Show Odum theoretical vs. measured SOA mass yield for all data points where the SQT:MT ratio was greater than 0.1. In (**b**) theoretical values were calculated assuming all terpene emissions could be represented with α-pinene yields. In (**c**) theoretical yields were calculated assuming the yields were a linear addition of α-pinene yields for monoterpenes and β-caryophyllene yields for sesquiterpenes.

Scots pine SOA yields were theoretically estimated using the Odum two-product approach^11^. Figure 5a shows the Odum curves for the two terpene standards that were oxidized in the Kuopio OFR. Odum fits are provided in the caption. Figure 5b and c show the theoretical versus measured SOA mass yield for all data points with an SQT:MT > 0.1 (average SQT:MT for these points was 0.27). This SQT:MT ratio is representative of typical unstressed emissions (refer to Fig. 2a). The theoretical yield was calculated assuming all terpenes exhibited vapor/condensed-phase partitioning as monoterpenes (5b) or that the final SOA mass yield was a linear addition of the monoterpenes and sesquiterpenes (5c). Monoterpene yields were estimated with α-pinene (aP) Odum fits and sesquiterpene yields were estimated with β-caryophyllene (bC) Odum fits. PTR-ToF-MS data was used to determine the fraction of monoterpene emissions relative to sesquiterpene emissions for the linear addition. When all terpenes were assumed to behave like monoterpenes, yields were under-predicted with a slope of 0.52. When sesquiterpenes were accounted for using a linear addition approach, the predicted values more closely matched the measured values with a slope of 0.87. This demonstrated that when SQT:MT ratios were similar to the typical unstressed pre-treatment values (see Fig. 1), the two-product model would under-predict SOA mass yields by approximately 50% if sesquiterpenes were neglected. When sesquiterpenes were accounted for, the theoretical predictions were greatly improved.

**Figure 6:**
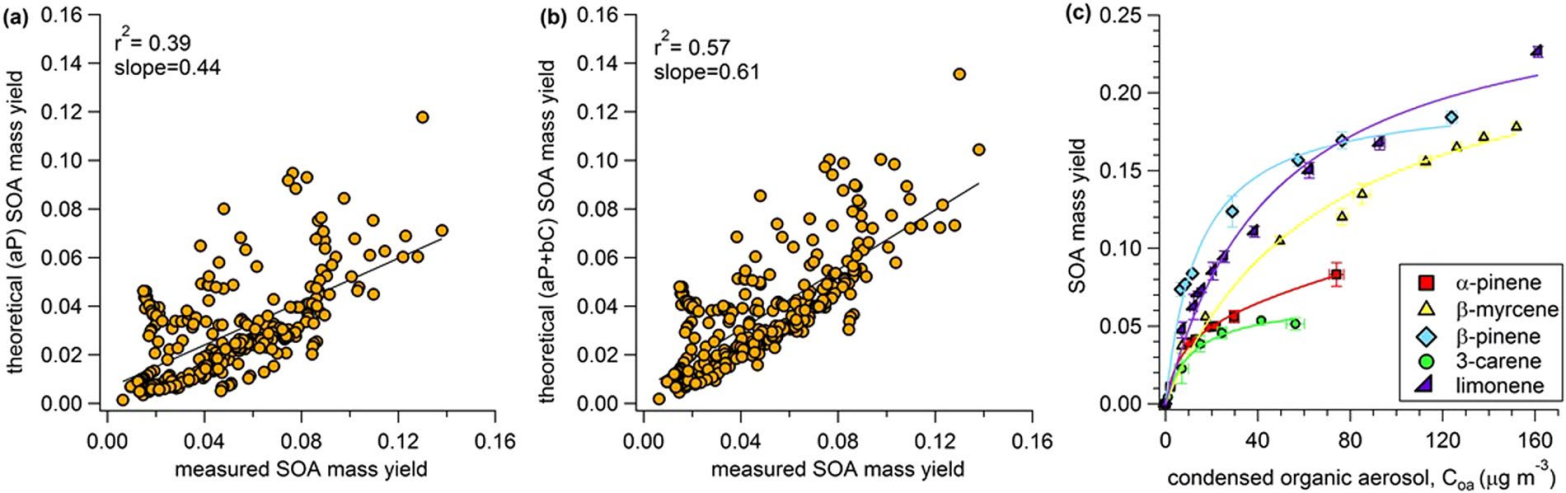
The influence of monoterpene chemical diversity on SOA mass yields. This figure shows Odum theoretical and observed SOA mass yields for the entire data-set. In (**a**) Odum theoretical yield was calculated assuming all terpenes had α-pinene yields and in (**b**) Odum theoretical yield was calculated assuming the yields were a linear addition of α-pinene yields for monoterpenes and β-caryophyllene yields for sesquiterpenes. (**c**) The range of SOA mass yields from oxidation of five different monoterpene standards that represent the major compounds emitted from Scots pines. Lines shown in (**c**) are Odum fits to the data.

SOA yields were not predicted as accurately for the entire data-set with all SQT:MT ratios (Fig. 6). Assuming all terpenes could be treated as monoterpenes, SOA yields were under-predicted by 66% (6a). Accounting for the relative amount of sesquiterpenes improved the estimate, but yields were still underpredicted by 49% (6b). This suggests that the different monoterpenes have different SOA mass yields and furthermore, representing all the monoterpene diversity with a single compound, such as the popular model compound α-pinene, is insufficient. This conclusion was supported by our measurements of SOA mass yield curves from five different monoterpene standards representing most of the dominant compounds in the plant emission profile (6c). At ~7 µg m^−3^ organic aerosol, the yield from β-myrcene was 0.023 while the yield from β-pinene was 0.074—more than three times greater than β-myrcene. Qualitatively, the monoterpene standard curves (Fig. 6c) suggest that SOA yields during active treatment in experiments 1 and 2 should have been higher than experiments 3 and 4 because the former had substantial limonene and β-myrcene emissions while the latter was dominated by 3-carene (refer to Fig. 3). This was generally true and is shown in the Supplementary information (S4). Yields during active feeding in experiments 1 and 2 were approximately double the yields during experiments 3 and 4. There were exceptions for the first 8 hours after active feeding started in experiment 4 when the yields were similar to experiments 1 and 2. Without detailed GC information corresponding to each SOA data point, we cannot investigate the influence of the monoterpene emission profile more quantitatively.

### Potential Impact on SOA Formation: Box Model Results

**Figure 7:**
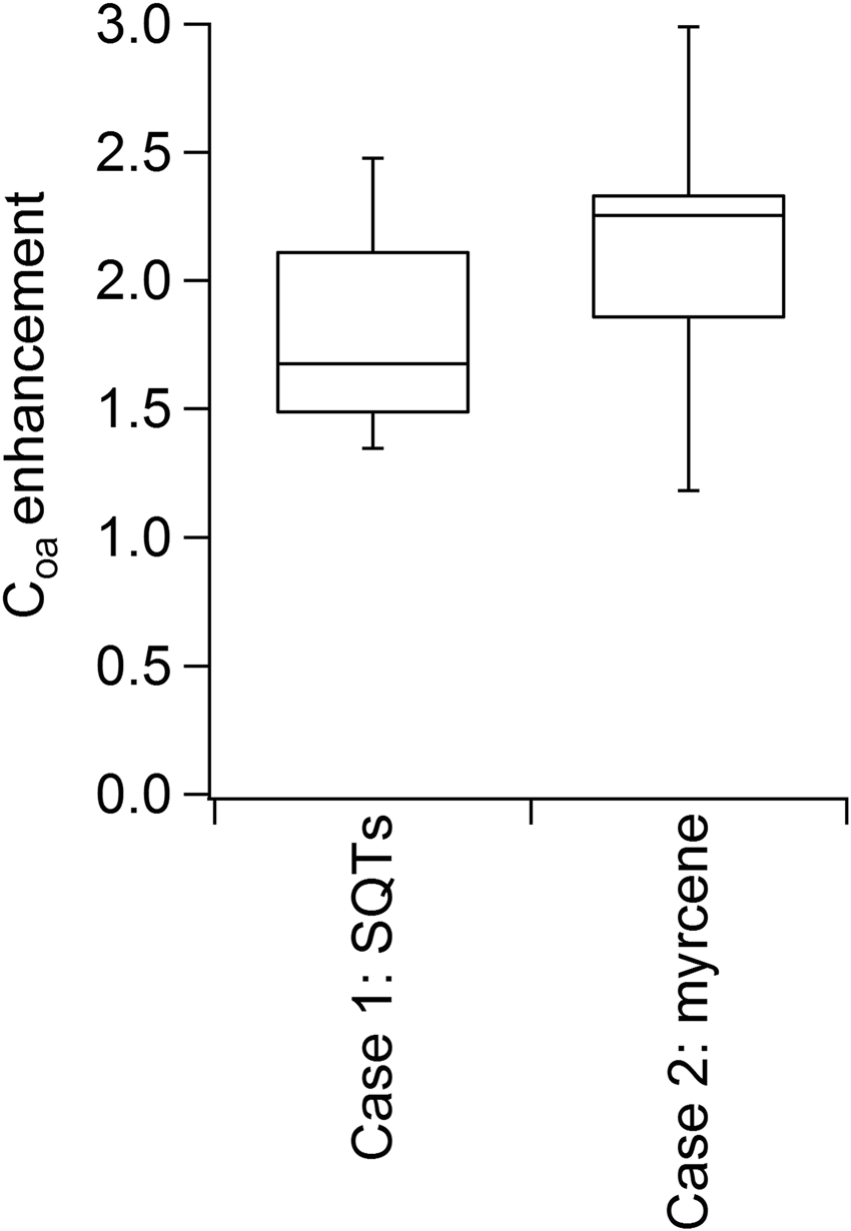
Organic aerosol enhancement from terpene complexity. Box model results illustrated enhancement of condensed organic aerosol formed for two cases. Enhancements calculated as the ratio of each case to the “α-pinene only base case”. Case 1: sesquiterpenes included and phase-partitioning was based on β-caryophyllene measurements. Case 2: monoterpene phase-partitioning was based on the β-myrcene Odum fit from measurements shown in Fig. 6c. Middle line denotes average, box edges denote 25^th^ and 75^th^ percentiles and bars denote 10^th^ and 90^th^ percentiles of enhancements calculated from MEGAN emissions using ICOS Sweden meteorological data May-August 2013 and 2014.

We used a box model to investigate the larger scale implications of simplifying terpene complexity for SOA production. We estimated organic aerosol mass in a boreal forest environment under three different partitioning scenarios—one base case and two test cases. The base case was an “α-pinene only” scenario and the two test cases represented (1) impacts of ignoring sesquiterpenes and (2) impacts of simplifying monoterpene diversity. In test case one, the sesquiterpenes were accounted for and phase-partitioning was treated as β-caryophyllene. In test case two, sesquiterpenes were excluded and monoterpene phase-partitioning was treated as β-myrcene. β-Myrcene was selected to contrast with α-pinene because it frequently comprised a major fraction of monoterpene emissions (Fig. 3) and had moderately higher SOA yields than α-pinene (Fig. 6c). The organic aerosol enhancement was calculated as the ratio of the total organic aerosol mass calculated for the test case divided by the base case. The enhancement is shown to compare the relative differences in SOA calculated using the Odum theory with different assumptions. This box model does not provide useful information about predicted SOA mass loadings because Odum fits generated in the OFR will not adequately represent gas-particle partitioning in a forest environment. However, comparing relative differences between different model assumptions provides meaningful results for the SOA modelling community. Further details about the box model calculation are included in the methods section. The results are shown in Fig. 7. The range of values reflects variability in estimates for environmental conditions representing May-August in 2013 and 2014 at the Svartberget ICOS Sweden station. The addition of sesquiterpenes to the box model enhanced SOA mass by 1.5 to 2.1-fold (25^th^–75^th^ percentile). This is likely a lower estimate because sesquiterpenes are highly reactive with ozone and only OH oxidation was used in the box model calculation. Using β-myrcene as a model monoterpene instead of α-pinene led to enhancements of organic aerosol of 1.9 to 2.3-fold (25^th^–75^th^ percentile). Again, this is likely a conservative estimate because many of the major monoterpene emissions from these Scots pines have higher SOA mass yields than β-myrcene.

## Discussion

There were two consistent herbivore stress responses from Scots pine seedlings in all four experiments. First, there was an overall increase in terpene emission rates. Second, there was a decrease in the SQT:MT ratio of the emission blend. These two observations have competing effects on SOA formation. Increased terpene emissions overall would provide more condensable organic material in the atmosphere, but the efficiency with which the organic material would condense to SOA would decrease. It is likely that the overall effect would be dominated by the increased emission rate and produce an increase in SOA because emissions increased by two orders of magnitude while SOA formation efficiency decreased by approximately half. Though outside the scope of this work, future studies should investigate these two competing effects from stressed Scots pine emissions in boreal forests using a regional chemical transport model.

Results from Figs 5 and 6 suggest that SOA formation from Scots pine emissions was controlled by (1) sesquiterpene emission rates in the healthy plants and (2) the blend of monoterpene isomers in the stressed plants. This is the first study to demonstrate the critical role of sesquiterpenes in SOA production by oxidizing plant volatile emissions directly. These results have important implications for SOA modeling. First, sesquiterpene emissions are likely underestimated in current inventories because they are relatively difficult to measure^41^. For example, a recent study of ambient volatiles in a pine forest using a new analytical approach reported many new sesquiterpenoid compounds that had not been reported previously^42^. Our results suggest that underestimating sesquiterpenes in the emissions inventory could have substantial impacts on SOA production. Additionally, even when emission models accurately simulate sesquiterpene emissions, their contributions to SOA in boreal forests are commonly ignored^8,9^ to simplify the chemical mechanisms in atmospheric models. The Model of Emissions from Gases and Aerosol from Nature (MEGAN) has a SQT:MT ratio for needleleaf boreal forests of 0.22 and our results clearly demonstrate that SOA yields could be underestimated by nearly 50% if the sesquiterpenes are ignored when SQT:MT > 0.1.

The other important result from Fig. 6 suggests that the specific blend of monoterpene emissions can also play a critical role in controlling SOA yields, particularly at SQT:MT < 0.1. This is an important result because α-pinene has often been used as a “model compound” to represent all monoterpenes for predictions of SOA formation from plant emissions, but SOA mass yields were underestimated by 39% (Fig. 6b) using α-pinene as a model compound for this data-set. While it is true that many of the state-of-the-art chemical transport models now include a few different monoterpenes (e.g. GEOS-chem), it is also true that (1) the monoterpene lumping approach used in these models is somewhat arbitrary and (2) other models, or simplified versions of the models, continue to use α -pinene as the biogenic SOA model compound (e.g. ECHAM-HAM or NorESM)^9,43,44^. Furthermore,  α-pinene is often still used as a model compound for laboratory studies investigating SOA behavior, for example chemistry in polluted atmospheres^36^, hygroscopicity and phase^5^, or evaporation kinetics^45^. Our results suggest that other monoterpenes, particularly limonene and β-myrcene, could be major contributors to SOA from Scots pine emissions and it would be prudent to start using these monoterpenes in laboratory investigations of SOA behavior and properties. We compared SOA mass yields from five different monoterpene isomers and demonstrated they had widely-varying SOA formation potential. Lumping all monoterpenes into a single category and treating them as α-pinene resulted in underpredicting SOA yield from the blend of terpenes studied here. Furthermore, these results emphasize that monoterpene lumping approaches could have a significant impact on final SOA results because the individual monoterpenes have widely-varying SOA yields. The same is likely true of sesquiterpenes. More effort should be made to identify the most important monoterpenes and sesquiterpenes in natural environments and to develop simplified lumping approaches that make the most sense for accurately predicting SOA generation.

The emissions data reported here add to the existing body of literature on pine weevil effects on boreal conifer emissions. There are a wide range of reported responses to pine weevil herbivory on boreal forest conifers. Heijari and colleagues (2011)^46^ reported a 2.8-fold and 2.9-fold systemic increase in monoterpene and sesquiterpene emissions, respectively, from the shoots of pine weevil damaged Scots pines. Thus, they observed no significant change in the relative SQT:MT ratio from healthy and herbivore-stressed plant shoot emissions. The SQT:MT ratio in shoot emissions from their study was ~0.017. Similarly, Joutsensaari and colleagues (2015)^25^ present emissions from control and herbivore-stressed plants after pine weevil herbivory. They found an SQT:MT of 0.02 and 0.005 in control and stressed Scots pine, respectively and an SQT:MT of 0.002 and 0.01 in control and stressed Norway spruce. Thus, exposure to pine weevil herbivores caused the SQT:MT ratio to either stay relatively constant^46^ or decrease^25^ in Scots pine, or to increase in Norway spruce^25^. However, in all plant stress cases, the SQT:MT ratio was less than 0.1. Our results demonstrated that the monoterpene diversity dominates SOA yields in cases where the emission mixture has SQT:MT < 0.1 (Figs 5 and 6). Thus our results suggest in all these stress emission cases the implications for SOA generation are likely dominated by the major monoterpene emission increases. This is even true in the case where Norway spruce sesquiterpene emissions exhibited a larger increase than monoterpene emissions because the final SQT:MT was 0.01. Overall, we conclude that SOA formation from boreal forest conifer emissions exposed to pine weevil herbivore stress would be controlled by the monoterpene emissions profile and that this should be the subject of future investigations on the topic. However, our results also suggest that SOA formation from healthy boreal forest conifers with SQT:MT ranging from 0.0–0.5^34^ could be substantially impacted by sesquiterpene emissions. These sesquiterpene emissions have often been ignored in SOA studies in boreal forests and should be another topic of future investigations.

In contrast to stress emissions from pine weevil herbivory, Zhao and colleagues (2017)^28^ exposed boreal forest trees to aphid herbivory and observed SQT:MT ratios of 0.119, 0.365 and 3.125 from healthy, intermediate stressed and maximum stressed plants, respectively. Similar to the plants in our study, Zhao and colleagues observed SQT:MT > 0.1 from healthy plant emissions, suggesting sesquiterpenes were significantly contributing to SOA yield before any stress. In contrast to our study, the aphid herbivore stress preferentially induced emissions of sesquiterpenes over monoterpenes leading to an increased SQT:MT ratio. Our results suggest in this case that SOA generation would be substantially enhanced due to the combined effects of (1) increasing overall emission rates and (2) drastically increasing the SOA mass yield of the mixture due to increased contribution from sesquiterpenes. Even though our observations after pine weevil herbivory had a drastically different effect on emissions, our conclusions regarding chemical controls on SOA generation are consistent with what was observed in Zhao *et al*.^28^.

Conservative box model estimates suggest that accounting for terpenoid complexity could enhance SOA mass loadings by 1.5- to 2.3-fold relative to an α-pinene model. Importantly, our calculations also demonstrated that representing the monoterpene diversity was just as important as accounting for the sesquiterpenes. These results provide key insights into areas for further study. In particular, more quantitative field measurements of sesquiterpene emission rates are needed with an emphasis on environmental controls, such as the fraction of light-dependent and light-independent emissions. Additionally, these results demonstrate that simplified chemical systems do not provide an adequate representation of SOA production from a more realistic mixture of plant volatile emissions. Future research should target SOA studies using plant volatile mixtures and detailed chemical measurements (including individual monoterpene isomer quantification) to identify the unifying molecular properties that drive SOA production from a mixture of terpenes. This will provide necessary information to simplify SOA models while still adequately representing the chemical complexity of the mixture.

## Methods

### Plant Description and Treatment

Plants were grown in 7.5-liter plastic pots in a 1:1:1 mixture of quartz sand, garden soil and natural peat in the Kuopio campus research garden at the University of Eastern Finland (UEF). Plants received fertilizer treatment (0.5 L of 0.1% fertilizer solution Turve-superex, N:P:K 12:5:27, Kekkilä Oy, Vantaa, Finland) once per week. Two plants were transported to the laboratory at least twenty-four hours before starting experiments. The plant lighting system consisted of four high output LED lamps directed at each plant (Valoya model B100, Valoya Oy, Helsinki Finland). Each plant was equipped with a dynamic enclosure secured to the trunk with cable ties—an ~70 liter custom-made Tedlar enclosure from Johnson Inert Products. ¼” PFA tubing were used for the inlet and outlet. The inlet tube carried approximately 4 lpm clean air to the enclosure and the outlet tube carried air from the enclosure to a three-way valve (Swagelok, Inc.) that was set to room air or to the OFR inlet. Plant enclosure outlet flow was pushed from the enclosure. Enclosure outlet flow rate was checked in-line daily to calculate final emission rates. Temperature was monitored in the enclosures with a thermocouple.

The adults of the large pine weevil (*Hylobius abietis* L.) used for the treatment were collected from pine sawdust storage at a sawmill (Iisveden Metsä Oy, Suonenjoki, Finland) and kept at +8 C. They were fed with freshly cut pine branches as a food source and starved for twenty-four hours prior to the start of experiments. The day of treatment, dynamic plant enclosures were carefully removed from both control and treatment plants. Four pine weevils were placed in a plastic foam enclosure (120 × 150 mm) with mesh sides and secured around the trunk of the treatment plant with clips. An empty foam enclosure was secured around the trunk of the control plant to account for any plant disturbance due to treatment application. Dynamic plant enclosures were carefully placed back on the control and treatment plants with the smaller mesh insect enclosure contained within the larger dynamic plant enclosure. Pine weevils were left on the treatment plant for forty-eight hours. Following insect enclosure removal, plant volatiles were monitored and SOA generation experiments were conducted for a minimum of three additional days.

### SOA Generation

Plant volatiles from Scots pines were photooxidized in a custom-built OFR. The OFR concept has been described in detail previously^37,38,47–49^. Prior to herbivore exposure, emissions from both the control and treatment plant were used for aerosol generation for a minimum of two days. This was necessary to get sufficient volatile mixing ratios for aerosol generation. After herbivore exposure, only the treatment plant was used for aerosol generation. All volatile measurements used to calculate SOA yield were measured directly from the OFR inlet. Flow through the OFR was controlled via suction from the analytical instruments and a vacuum mass flow controller. OFR flows ranged from 2.5–3.6 lpm with corresponding residence times of 60–90 seconds. OH radicals were generated in the flow reactor from a combination of ozone, water vapor and high intensity mercury lamps (LT 36 W/UV-C, Sylvani Germicidal linear, Germany). Ozone was generated with a calibration source (part number UVP SOG-2). Ozone mixing ratios at the OFR inlet were approximately 450 ppb. RH was maintained in the OFR between 30–40% using humidified flow generated with a Nafion membrane humidifier (Perma Pure FC-Series humidifier, model FC-125-240-5PP). The plant volatile line was connected to a clean dilution air source, a three-way valve connected to the plant enclosure and an exhaust line located downstream of the three-way valve. The plant volatile flow was mixed with variable levels of dilution air to generate SOA efficiency curves at multiple mass loadings.

For comparison purposes, monoterpene and sesquiterpene standards were also used to generate SOA efficiency curves. Standards were introduced with a glass diffusion bottle containing a small vial of a standard compound. The standard monoterpenes used were α-pinene (Sigma-Aldrich, ≥99%), 3-carene (Sigma-Aldrich, ≥95%), β-myrcene (Sigma-Aldrich, ≥90%), β-pinene (Sigma-Aldrich, ≥99%), limonene (Sigma-Aldrich, ≥97%) and the standard sesquiterpene used was β-caryophyllene (Sigma-Aldrich, ≥98.5%). All flows were controlled with mass flow controllers unless otherwise noted (AliCat Scientific, Inc.). OH exposures in the OFR ranged from 6.6 × 10^10^–3.3 × 10^11^ molec cm^−3^ s based on offline calibrations with SO_2_ as described in Lambe *et al*.^37^. This corresponds to an approximate photochemical age from 0.5–2.5 days assuming [OH] = 1.5 × 10^6^ molec cm^−3^. Note that these have not been corrected for external OH reactivity and thus represent an upper bound for OH exposure^49^.

### Instruments and Calculations

Plant volatile emissions were monitored semi-continuously from the control and treatment plant with a proton transfer reaction time of flight mass spectrometer, PTR-ToF-MS (Ionicon, Inc., PTR-ToF 8000). The PTR-ToF-MS was connected to an automated valve switching system that alternated between control tree enclosure, treatment tree enclosure (used as OFR inlet measurement for active feeding and post-treatment points), OFR inlet (for pre-treatment points only) and OFR outlet with 20 minutes of sampling at each location. Details about PTR-ToF-MS data processing are described in detail in the supplement. PTR-ToF-MS measurements were supplemented approximately twice daily (morning and afternoon) with duplicate adsorbent cartridge samples (Tenax TA adsorbent). Cartridges were analyzed via thermo-desorption gas chromatograph mass spectrometry, TD-GC-MS (TD: Perkin Elmer, ATD 400, USA; GC-MS: Hewlett Packard, GC 6890, MSD 5973, USA). The following compounds were resolved and quantified with authentic GC standards: α-pinene, camphene, β-pinene, β-myrcene, 3-carene, limonene, longifolene, β-caryophyllene, and β-farnesene. P-Cymene and β-phellandrene were also dominant contributors to the emission profile, but did not have a corresponding authentic standard. In that case, terpinolene was used as a proxy standard for their quantification. In addition, 23 other monoterpenoids and 13 sesquiterpenes were included in the “other” categories. These compounds are listed in the supplementary information in the footnote of Table S1. A comprehensive list of the compounds included in the authentic standard are provided in the supplementary information. All plant volatile measurements were reported as Basal Emission Rates (BER)— normalized to total needle surface area and to a standard temperature of 303 K. Temperature-normalization was performed using the Guenther algorithm with β = 0.09 for monoterpenes and β = 0.17 for sesquiterpenes^1^. Needle surface area was approximated via the methods presented in Kivimäenpää *et al*.^21^, following Flower-Ellis and Olsson^50^. A comparison of PTR and GC measurements is presented in the supplementary information (Figure S3). PTR-ToF-MS sesquiterpene measurements were corrected for sampling line wall losses based on this inter-comparison.

SOA mass yields (Y) were calculated from the amount of organic aerosol mass generated in the OFR divided by the mass of terpenoids that reacted (monoterpenes + sesquiterpenes).

1 $$Y=\frac{{\Delta }{C}_{OA}}{{\Delta }VO{C}_{MT+SQT}}$$

Where ΔC_OA_ is the condensed organic aerosol mass formed in the flow reactor (µg m^−3^) and the ΔVOC_MT+SQT_ is the mass of total monoterpenes and sesquiterpenes that reacted in the flow reactor (µg m^−3^). Organic aerosol mass was calculated from particle size distribution measurements with a scanning mobility particle sizer, SMPS (TSI, Inc., Model DMA 3082, CPC 3775). Volume distributions were converted to mass using the SOA density calculated from a comparison of aerodynamic median diameter measured with the high resolution time of flight aerosol mass spectrometer, HR-ToF-AMS (Aerodyne, Inc) and the median mobility diameter measured with the SMPS. SOA densities ranged from 1.1–1.4 g cm^−3^. When size distributions were too small to obtain appreciable signal with the AMS, the average value of 1.2 g cm^−3^ was used. This is consistent with other observations of the density of biogenic SOA^51,52^. Mass of reacted gas-phase terpenoids was calculated from PTR-ToF-MS measurements made at the inlet and outlet of the OFR. All terpenoids reacted in the OFR for the conditions in these experiments. Additional measurements at the OFR outlet included ozone mixing ratios (Thermo, Model 49i) and RH and temperature (Vaisala HMP110).

### Theoretical SOA Yields

Theoretical SOA mass yields were calculated using the Odum 2-product approach^11^. Briefly, the SOA mass yield is expressed by the following equation:

2 $${\rm{Y}}={C}_{OA}{\sum }_{i}\frac{{\alpha }_{i}{K}_{i}}{1+{K}_{i}{C}_{OA}}$$

Where C_OA_ is the total condensed organic aerosol mass in the flow reactor (µg m^−3^), α_i_ is the proportionality constant relating the amount of VOC that reacted to the total concentration of product, i and K_i_ is the partitioning coefficient for species, i. We fit the yield curves generated from standard compounds (and shown in Figs 5a and 6c) using equation (2) assuming two species. The fitted parameters used in the theoretical calculations are shown in Fig. 5. These fit parameters were then used to calculate the theoretical yield from measured organic aerosol masses for each Scots pine SOA data point using equation (2). Theoretical yields for two scenarios were calculated. Scenario 1 (aP): all condensing vapors were treated as α-pinene. Scenario 2 (aP + bC): the yield was a linear addition of the monoterpene and sesquiterpene emissions where monoterpenes were treated as α-pinene and sesquiterpenes were treated as β-caryophyllene.

### Box Model Calculation

Monoterpene and sesquiterpene emission rates were estimated from a boreal forest using a simplified Excel version of MEGAN v2.1^1^. The emission type was “needleleaf evergreen boreal”. Meteorological inputs for the model were obtained from the ICOS Sweden Svartberget station (64°15′N, 19°46′E) for May-August 2013 and 2014. The Svartberget station is in a boreal forest with primarily Scots pine (*Pinus sylvestris*) and Norway spruce (*Picea abies*) vegetation. Other MEGAN inputs included: leaf area index = 5 and vegetation cover fraction = 1. The influence of temperature and light for the past 24 hours and 240 hours was accounted for. Model output was hourly monoterpene and sesquiterpene emission rates (µg m^−2^ s^−1^). Emission rates were used to estimate an approximate hourly atmospheric concentration (µg m^−3^) assuming a PBL height of 1 km and lifetimes based on OH reaction rate constants^53^ (α-pinene, k_OH_ = 5.37 × 10^−11^ cm^3^ molecule^−1^ s^−1^ or β-myrcene, k_OH_ = 2.15 × 10^−10^ cm^3^ molecule^−1^ s^−1^ for monoterpenes and β-caryophyllene, k_OH_ = 1.97 × 10^−10^ cm^3^ molecule^−1^ s^−1^ for sesquiterpenes). [OH] concentration was set to 1 × 10^6^ molecules cm^−3^. Total organic aerosol produced from the reacted VOCs was calculated with the following equation:

3 $${C}_{OA}={C}_{OA,b}+{\sum }_{i}({Y}_{i}\,\ast \,VO{C}_{r,i})$$

where C_OA,b_ is the initial background concentration of condensed organic mass (assumed to be 1 µg m^−3^), VOC_r,i_ is the mass of reacted VOCs for species, i (µg m^−3^) and Y_i_ is the yield for species i calculated from equation (2) and assuming a background C_OA_ of 1 µg m^−3^.

### Data availability

The data supporting the findings of this study are available upon reasonable request to the corresponding author.

## Electronic supplementary material

Supplementary Information
