# Supplementary material for: Terpene Composition Complexity Controls Secondary Organic Aerosol Yields from Scots Pine Volatile Emissions
Source: Sci Rep. 2018 Feb 14;8:3053. doi: 10.1038/s41598-018-21045-1 (PMC5813208; doi:10.1038/s41598-018-21045-1)
Supplement: Supplementary file 1 — Supplementary Information [file 41598_2018_21045_MOESM1_ESM.pdf]

Supplementary Information for: **Terpene Composition Complexity Controls Secondary Organic Aerosol Yields from Scots Pine Volatile Emissions**

Faiola, C.L.[1,2*], Buchholz, A.[1], Kari, E.[1], Yli-Pirilä, P.[3], Holopainen, J.K.[3], Kivimäenpää, M.[3], Miettinen, P.[1], Worsnop, D.R.[1,4,5], Lehtinen, K.E.J.[1,6], Guenther, A.B.[7], and Virtanen, A.[1]

[1]Department of Applied Physics, University of Eastern Finland, P.O. Box 1626, 70211 Kuopio, Finland

[2]Department of Ecology and Evolutionary Biology, University of California Irvine, Irvine, CA 92697-2525, United States

[3]Department of Environmental and Biological Sciences, University of Eastern Finland, P.O. Box 1627, 70211 Kuopio, Finland

[4]Aerodyne Research Inc., Billerica, Massachusetts 08121-3976, United States

[5]Department of Physics, University of Helsinki, P.O. Box 64, 00014 Helsinki, Finland

[6]Finnish Meteorological Institute, Kuopio, PO Box 1627, 70211 Kuopio, Finland

[7]Department of Earth System Science, University of California, Irvine, CA 92697-3100, United States

*Correspondence and request for materials should be addressed to Celia Faiola (cfaiola@uci.edu).

PTR-ToF-MS Data Processing

The mass scale calibration of the PTR-ToF-MS was performed using 3 compounds that were always present in mass spectra of the tree emissions: $H_3O_{18}^+$ at m/z 21.0226, NO^+ at m/z 29.998, and $C_{10}H_{17}^+$ at m/z 137.133. Furthermore, the transmission efficiency of ions with different molar masses through the PTR-ToF-MS was calibrated using a calibration gas standard containing 8 aromatic compounds with mixing ratios ~100 ppbV in nitrogen (BOC, United Kingdom). The mass scale calibration and peak fitting for PTR-ToF-MS data were done using PTR-MS Viewer software (Ionicon Analytik), and the data were further analyzed using MATLAB. For PTR-ToF-MS data-analysis of monoterpenes and sesquiterpenes, the calibration corrected molecular ions were used to calculate the emission rates of these compounds. Therefore, for monoterpenes the peak present at m/z 137.13 was used, and for sesquiterpenes the peak at m/z 205.20. The calibration of the PTR-ToF-MS with pure standards was done using a dynamic dilution system (Faiola et al., 2012; Kari et al., in review). From the calibration of pure standards the calibration factors of molecular ions of mono- and sesquiterpenes for PTR-ToF-MS in this study were calculated using the weighted averages based on GC-MS data. For monoterpenes the calibration factor used was the weighted average of the calibration factors of following compounds: α-pinene, camphene, β-myrcene, β-pinene, 3-carene, δ-limonene, and β-phellandrene for which all, except for camphene and β-phellandrene, the calibration was done using the pure compounds. For camphene and β-phellandrene the calibration factors of β-pinene and limonene was used, respectively, because of the similar structures the compounds have. The calibration factor used to correct the molecular ion of sesquiterpenes was calculated from the calibration factors of longifolene, β-caryophyllene, α-muurolene, α-amorphene, and δ-cadinene. For longifolene, and β-caryophyllene the pure standards were used for the determination of the calibration factors. For the rest of sesquiterpenes the calibration factor of α-humulene was used.

GC-MS Authentic Standards

An authentic terpenoid and green leaf volatile standard was run with each set of GC cartridge samples. Two standard cartridges were prepared with each standard. The standard contained the following compounds: α-pinene, camphene, sabinene, β-pinene, β-myrcene, 3-carene, limonene, 1,8-cineole, γ-terpinene, terpinolene, linalool, E-DMNT, camphor, borneol, terpinen-4-ol, α-terpineol, bornyl acetate, longifolene, trans-β-farnesene, α-humulene, α-copaene, trans-caryophyllene, aromadendrene, β-elemene. The green leaf volatile standard contained the following compounds: cis-3-hexen-1-ol, trans-2-hexenal, 1-hexanol, 1-octen-3-ol, cis-3-hexenyl acetate, 1-chloro-octane, nonanal, cis-3-hexenyl butyrate, methyl salicylate, cis-3-hexenyl isovalerate, and cis-3-hexenyl tiglate. A stock solution of each mixture was prepared in 100 mL of methanol. The stock solution was diluted in methanol 10:1 and 2 μL were injected onto adsorbent cartridges with a micro-syringe.

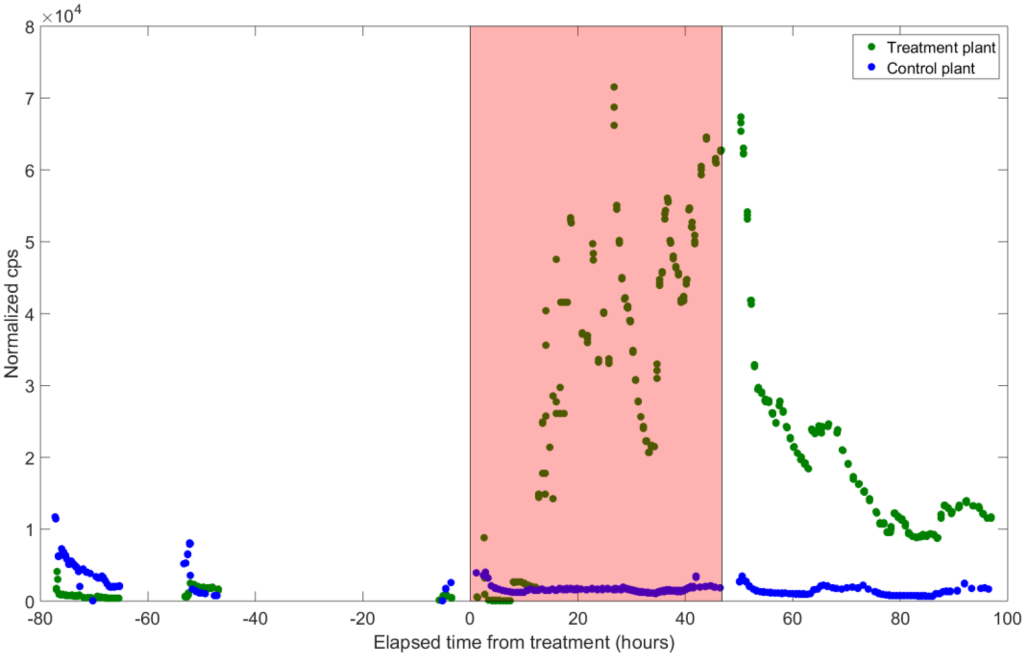

Figure S1: A time-series from experiment 1 of normalized counts per second (cps) for m137, the molecular ion of monoterpenes. The data was corrected with a calibration factor to account for fragmentation in the PTR-ToF-MS. The shaded region denotes the herbivore exposure. The m137 measured from the treatment plant emissions is shown in green and from the control plant is shown in blue. Note the delay in emission increase after herbivores were applied. An

herbivore emission effect was not observed until after the herbivores began feeding the following morning.

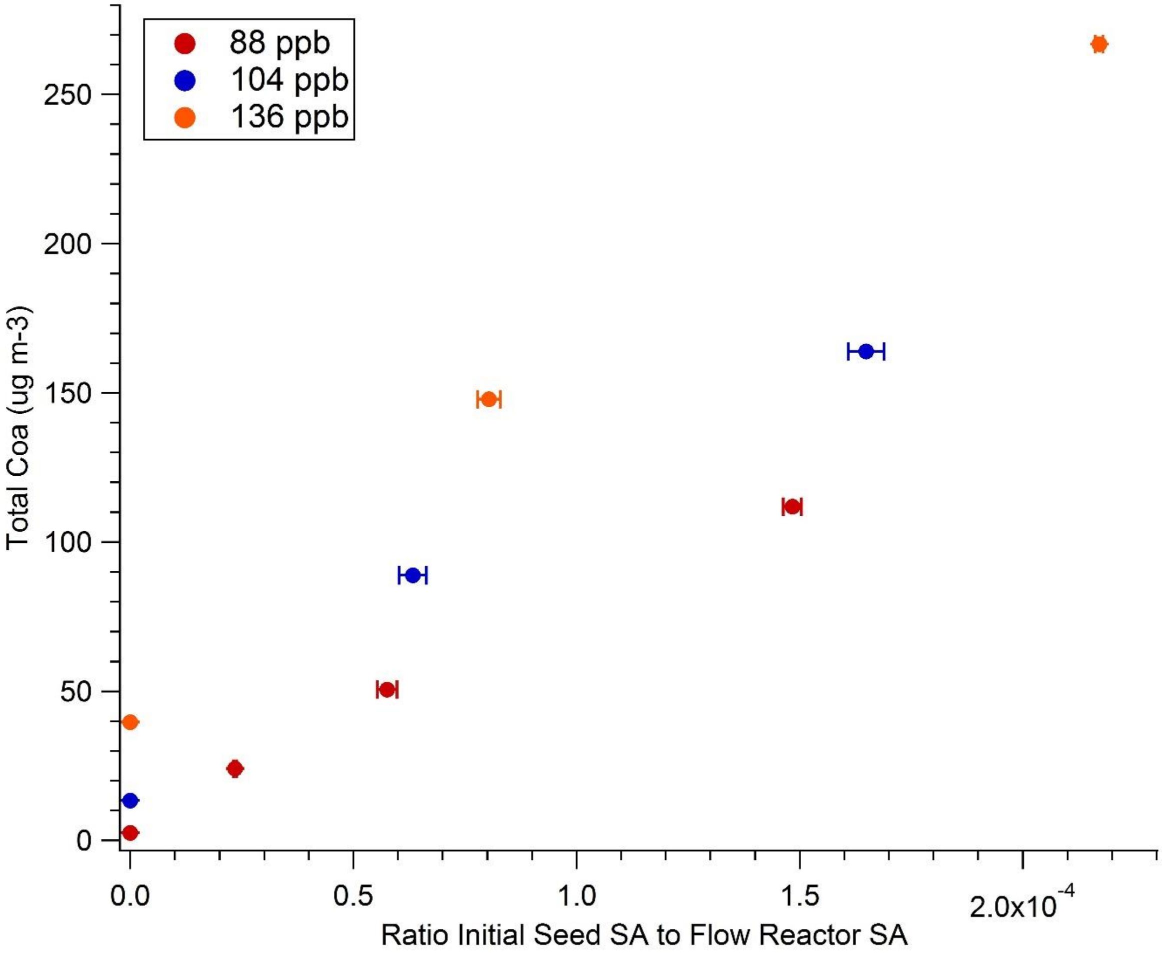

Figure S2: SOA mass yield versus the ratio of the initial seed particle surface area ($S_{s,i}$) to the flow reactor surface area (S_f) for three different α-pinene mixing ratios. Yields were strongly dependent on seed surface area. For example, SOA mass yield from 88 ppb reacted α-pinene increased from 1% without any seed to nearly 11% with high seed.

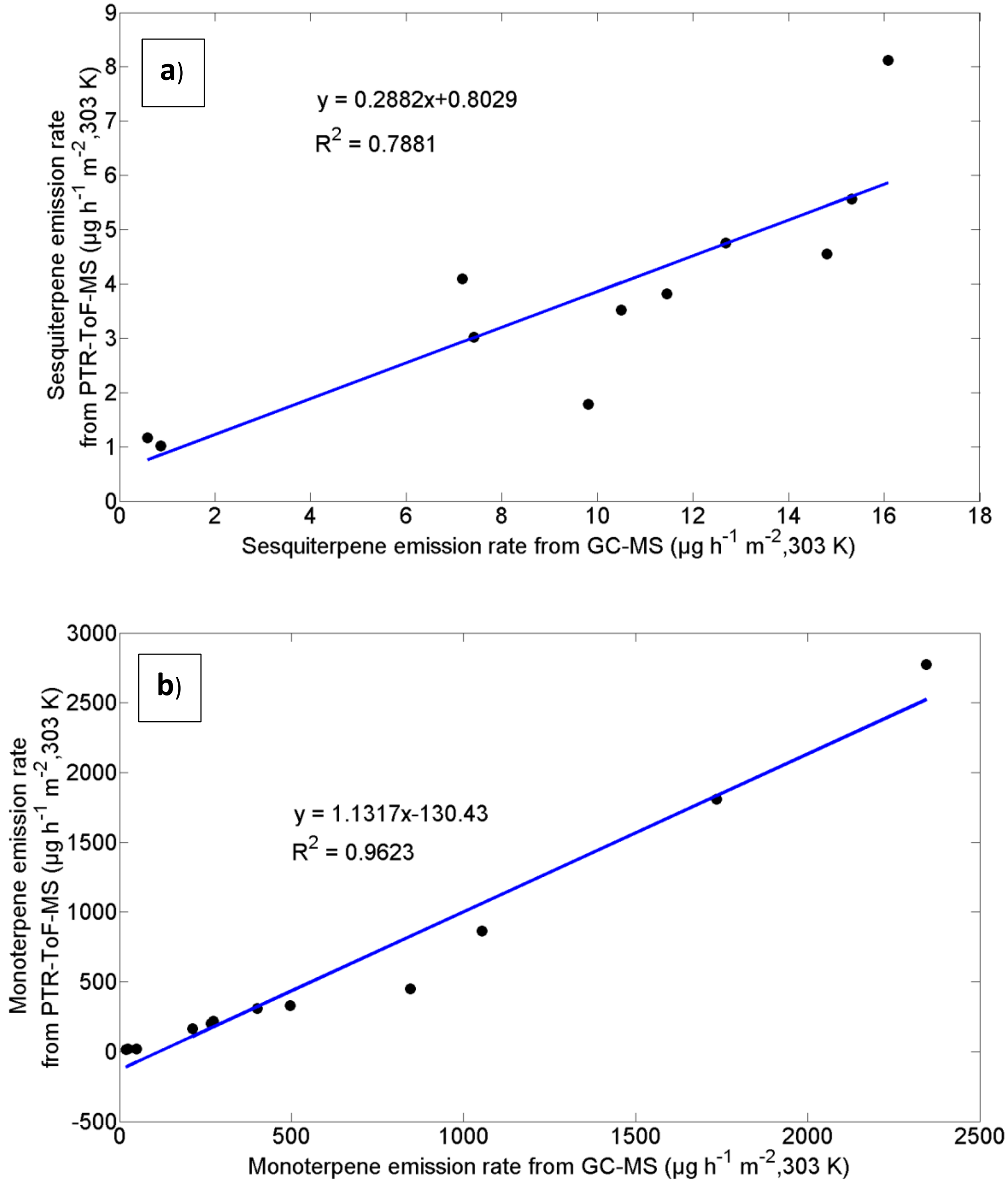

Figure S3: Inter-comparison between PTR-ToF-MS and TD-GC-MS measurements for sesquiterpene emissions (a), and monoterpene emissions (b). TD-GC-MS cartridge samples were collected immediately downstream of the plant enclosure to minimize sampling line losses. The PTR was connected to an automated valve system with long sampling lines. There were minimal sampling line losses of monoterpenes, and high losses of sesquiterpenes. PTR sesquiterpene concentrations were corrected for loss by multiplying PTR values by 3.27 based on the slope in (a). Sesquiterpenes were also corrected for fragmentation patterns from standard runs (Kari et al., in preparation).

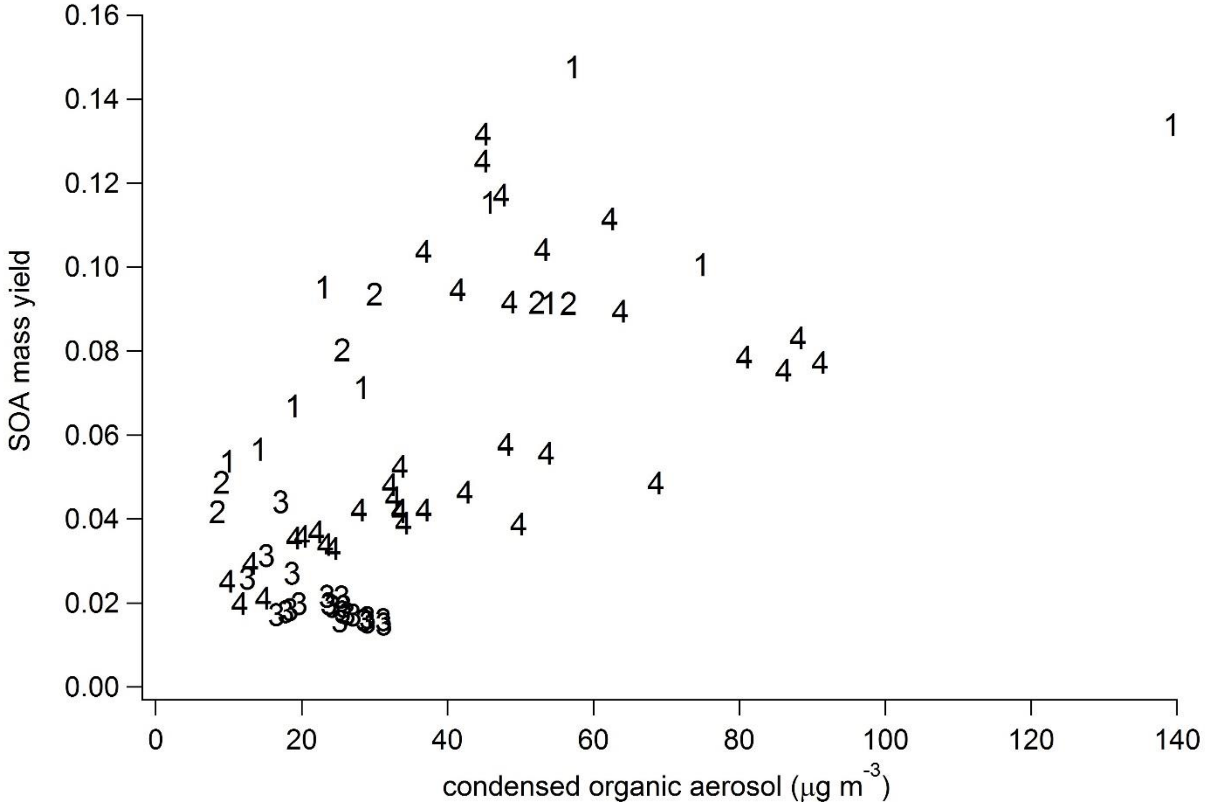

Figure S4: SOA mass yields during active feeding periods. Marker numbers denote the experiment number. Generally, SOA mass yields were higher during active feeding periods in experiments 1 and 2 than they were in experiments 3 and 4.

Table S1: Average basal emission rates (μg m^{-2} h^{-1}, 303 K) of plant volatiles during each experimental phase (pre-treatment, active feeding, and post-treatment) for each experiment. Errors are provided in parentheses and were propagated from the standard deviations of duplicate cartridge samples analyzed via TD-GC-MS. bdl=below detection limit.

		**Pre-Treatment**		**Active**		**Post-Treatment**	
	Exp #	Control	Treatment	Control	Treatment	Control	Treatment
α-pinene	1	30.8 (4.9)	4.2 (0.3)	20.2 (1.0)	247.9 (20.8)	1.2 (0.1)	13.1 (0.4)
	2	15.0 (0.4)	8.5 (0.1)	6.0 (0.3)	462.9 (34.2)	6.7 (0.4)	431.4 (6.8)
	3	2.9 (0.3)	2.3 (0.1)	2.3 (0.09)	164.1 (24.0)	1.8 (0.04)	57.8 (6.5)
	4	8.1 (0.3)	9.6 (0.7)	8.0 (0.2)	239.1 (7.9)	3.8 (0.2)	32.1 (2.2)
camphene	1	12.3 (1.9)	1.7 (0.1)	6.5 (1.1)	292.2 (56.1)	0.3 (0.05)	6.9 (0.3)
	2	3.2 (0.3)	6.1 (0.03)	1.3 (0.3)	239.9 (19.4)	0.7 (0.06)	75.7 (2.4)
	3	1.9 (0.2)	0.9 (0.03)	1.7 (0.1)	84.0 (8.3)	1.3 (0.03)	18.1 (1.9)
	4	2.7 (0.1)	3.2 (0.3)	0.9 (0.05)	9.6 (3.5)	0.9 (0.1)	6.9 (0.7)
β-pinene	1	1.6 (0.1)	1.4 (0.1)	0.9 (0.05)	134.1 (16.6)	0.07 (0.004)	0.8 (0.02)
	2	0.7 (0.03)	2.0 (0.02)	0.8 (0.2)	128.2 (14.4)	0.4 (0.04)	108.4 (2.5)
	3	0.5 (0.03)	0.4 (0.01)	0.4 (0.02)	12.5 (2.7)	0.4 (0.05)	3.1 (0.3)
	4	0.6 (0.02)	0.9 (0.08)	0.4 (0.03)	10.3 (1.3)	0.2 (0.01)	1.2 (0.06)
β-myrcene	1	1.9 (0.3)	1.3 (0.02)	1.3 (0.1)	564.0 (63.6)	0.08 (0.01)	2.9 (0.06)
	2	1.3 (0.08)	1.0 (0.01)	2.6 (0.8)	123.2 (2.2)	1.7 (0.06)	71.1 (0.7)
	3	0.6 (0.03)	0.5 (0.02)	0.5 (0.02)	36.1 (2.2)	0.4 (0.02)	6.2 (0.5)
	4	1.5 (0.08)	17.6 (1.4)	0.5 (0.02)	63.5 (3.4)	0.2 (0.01)	3.6 (0.2)
Δ-3-carene	1	26.7 (2.0)	bdl	16.8 (0.8)	2.0 (0.4)	1.1 (0.07)	0.03 (0.003)
	2	6.8 (0.2)	bdl	6.4 (1.5)	1.2 (0.05)	3.3 (0.07)	0.3 (0.2)
	3	7.4 (0.5)	5.7 (0.1)	4.6 (0.1)	448.1 (27.8)	3.4 (0.02)	129.4 (8.0)
	4	9.8 (0.4)	22.8 (1.9)	4.6 (0.1)	302.6 (12.9)	1.6 (0.05)	33.9 (1.0)
p-cymene*	1	13.6 (5.2)	1.1 (0.5)	25.1 (4.4)	1584.2 (280.8)	10.0 (6.7)	47.3 (28.8)
	2	16.7 (10.1)	15.5 (12.1)	7.5 (3.6)	766.7 (109.9)	0.9 (0.06)	305.3 (50.0)
	3	16.1 (10.9)	12.4 (6.5)	7.6 (5.3)	185.8 (62.2)	1.5 (0.3)	62.7 (11.4)
	4	5.2 (2.5)	4.2 (0.9)	0.3 (0.1)	10.9 (4.0)	1.1 (0.4)	11.7 (3.2)
limonene	1	1.9 (0.3)	1.3 (0.1)	1.0 (0.2)	356.3 (48.9)	0.06 (0.01)	4.6 (0.2)
	2	0.6 (0.09)	3.0 (0.09)	0.4 (0.09)	538.4 (28.6)	0.3 (0.03)	186.7 (3.6)
	3	0.2 (0.02)	2.3 (0.08)	0.2 (0.03)	115.6 (9.2)	0.1 (0.05)	17.7 (1.9)
	4	7.1 (0.3)	1.4 (0.2)	2.2 (0.1)	22.5 (7.9)	0.8 (0.04)	2.2 (0.3)

β-phellandrene*	1	0.6 (0.09)	0.1 (0.1)	0.5 (0.03)	26.5 (3.6)	bdl	0.3 (0.01)
	2	0.8 (0.08)	1.1 (0.01)	2.3 (0.7)	212.4 (1.4)	1.2 (0.01)	134.5 (2.2)
	3	0.4 (0.02)	2.2 (0.09)	0.2 (0.01)	102.6 (8.6)	0.2 (0.02)	11.3 (0.8)
	4	13.5 (0.7)	22.6 (2.0)	5.1 (0.2)	138.4 (4.3)	1.2 (0.06)	8.1 (0.3)
Other MT**	1	9.0 (0.5)	1.3 (0.1)	4.9 (0.4)	705.7 (58.4)	0.3 (0.02)	9.0 (0.4)
	2	3.5 (0.4)	3.1 (0.08)	2.1 (0.3)	296.4 (18.3)	1.7 (0.1)	105.7 (2.7)
	3	7.4 (0.4)	4.6 (0.2)	7.1 (0.3)	259.0 (8.6)	2.2 (0.01)	36.1 (0.8)
	4	4.3 (0.6)	7.5 (0.2)	1.5 (0.03)	92.3 (5.3)	1.0 (0.05)	10.4 (0.4)
longifolene	1	0.4 (0.002)	bdl	0.5 (0.03)	0.1 (0.1)	bdl	bdl
	2	0.7 (0.02)	0.2 (0.1)	0.6 (0.02)	0.3 (0.01)	0.4 (0.005)	0.3 (0.01)
	3	0.1 (0.007)	0.2 (0.01)	0.1 (0.007)	0.9 (0.03)	0.07 (0.07)	0.6 (0.01)
	4	0.3 (0.01)	0.1 (0.006)	0.2 (0.004)	0.2 (0.009)	0.1 (0.006)	0.09 (0.004)
β-caryophyllene	1	2.7 (0.3)	bdl	1.4 (0.1)	0.6 (0.04)	0.06 (0.006)	0.05 (0.003)
	2	1.5 (0.07)	0.5 (0.01)	0.3 (0.02)	2.7 (0.2)	0.1 (0.01)	1.1 (0.02)
	3	0.1 (0.06)	bdl	0.1 (0.003)	0.1 (0.007)	0.2 (0.01)	0.1 (0.02)
	4	0.5 (0.03)	1.8 (0.09)	0.3 (0.008)	1.5 (0.09)	0.02 (0.009)	0.1 (0.005)
β-farnesene	1	bdl	0.7 (0.05)	bdl	2.3 (0.2)	bdl	0.1 (0.01)
	2	1.7 (0.8)	2.1 (0.02)	bdl	bdl	bdl	bdl
	3	0.1 (0.02)	0.2 (0.01)	0.9 (0.2)	1.1 (0.1)	bdl	0.1 (0.01)
	4	2.4 (0.1)	1.6 (0.2)	0.3 (0.013)	0.2 (0.01)	bdl	0.05 (0.005)
Other SQT**	1	38.2 (0.6)	3.6 (0.1)	24.3 (1.0)	20.8 (0.6)	1.2 (0.04)	2.9 (0.06)
	2	31.2 (0.7)	18.5 (0.1)	8.7 (0.2)	70.6 (2.5)	6.7 (0.3)	56.4 (1.0)
	3	5.7 (0.3)	0.8 (0.1)	4.3 (0.2)	9.2 (0.2)	3.2 (0.01)	9.3 (0.1)
	4	3.9 (0.1)	8.7 (0.2)	2.6 (0.05)	15.2 (0.6)	0.7 (0.03)	7.5 (0.2)

*These compounds were not included in the GC standard and thus these BER values are semi-quantitative. MS quantitation was performed using the terpinolene standard as a proxy. Terpinolene was selected as a proxy because of similarity in structure to β-phellandrene.

**Many of the compounds in the "other" categories were not included in the GC standard. A proxy was chosen from the standard compounds based on structural and mass spectral similarity. These BER values are also semi-quantitative. Compounds in the other monoterpenoids category include the following: tricyclene, β-fenchene, α-fenchene, sabinene, α-phellandrene, α-terpinene, 1,8-cineole, β-ocimene, γ-terpinene, terpinolene, camphor, borneol, bornyl acetate, and 10 unidentified monoterpenes. The unidentified monoterpenes had clear monoterpene mass spectra, but NIST identification matched them with monoterpenes that had known different retention times. Compounds in the other sesquiterpenes category include the following: α-copaene, β-elemene, junipene, isolongifolene, α-humulene, β-cubebene, valencene, murolene,

α-amorphene, δ-cadinene, cadina-1,4-diene, and two unidentified compounds that clearly had a sesquiterpene NIST spectra match, but the match was not consistent with other known sesquiterpene retention times.
